# Chemical shift assignment of a thermophile frataxin

**DOI:** 10.1007/s12104-017-9790-3

**Published:** 2017-10-31

**Authors:** Masooma Rasheed, Robert Yan, Geoff Kelly, Annalisa Pastore

**Affiliations:** 1 Maurice Wohl Institute, King’s College London, 5 Cutcombe Rd London, SE5 9RT UK; 2 MRC-NMR Centre, The Crick Institute, London, NW7 1AT UK; 3 Molecular Medicine Department, University of Pavia, Pavia, Italy

**Keywords:** Frataxin, Friedreich ataxia, Iron–sulfur cluster, Metal binding proteins, Structure

## Abstract

Frataxin is the protein responsible for the genetically-inherited neurodegenerative disease Friedreich’s ataxia caused by partial silencing of the protein and loss of function. Although the frataxin function is not yet entirely clear, it has been associated to the machine that builds iron–sulfur clusters, essential prosthetic groups involved in several processes and is strongly conserved in organisms from bacteria to humans. Two of its important molecular partners are the protein NFS1 (or IscS in bacteria), that is the desulfurase which converts cysteine to alanine and produces sulfur, and ISU (or IscU), the scaffold protein which transiently accepts the cluster. While bacterial frataxin has been extensively characterized, only few eukaryotic frataxins have been described. Here we report the ^1^H, ^13^C and ^15^N backbone and side-chain chemical shift assignments of frataxin from *Chaetomium thermophilum*, a thermophile increasingly used by virtue of its stability.

## Biological context

Iron sulfur (FeS) clusters are important protein cofactors present in all organisms and involved in structural and functional roles thanks to their favorable redox potential (Zanello 2017; Rouault and Maio 2017). Synthesis and assembly of FeS clusters in proteins is under the control of evolutionary conserved machines (Maio and Rouault 2015; Blanc et al. 2015). One of the proteins involved in the process is frataxin, a protein whose reduced levels cause in humans the neurodegenerative disease Friedreich’s ataxia (Pastore and Puccio 2013). This is a genetic condition which leads to loss of voluntary muscle movement, diabetes and eventually causes death from cardiac complications. Frataxins are iron binding proteins which are found in organisms as diverse as bacteria and eukaryotes up to humans (Gibson et al. 1996). They contain a highly conserved globular C-terminal domain in eukaryotes preceded by an N-terminal tail which is needed for import of the protein to the mitochondria, where most of the frataxin functions occur (Braymer and Lill 2017). The structures of bacterial, yeast and human frataxins have been reported (Musco et al. 2000; Nair et al. 2004; Karlberg et al. 2006; Roman et al. 2013). Amongst the frataxin partners are the desulfurase NFS1 (IscS in prokaryotes) and the scaffold protein ISU (IscU in prokaryotes), which are essential components of the FeS cluster machine and form a ternary complex with frataxin (Prischi et al. 2010; Shi et al. 2010). Understanding the specific role that frataxin has in FeS cluster biogenesis is an important goal which would help to design treatments of the pathology. Yet, to date, no structure of the quaternary NFS1–ISU–ISD11–frataxin complex is known.

Here, we report the practically complete assignment of the backbone of an *Escherichia coli* expressed evolutionary conserved C-terminal frataxin domain from the fungus *Chaetomium thermophilum* (Ct) with the ultimate goal of using it for further binding studies. The use of proteins from this more stable thermophile has recently been adopted as a source of proteins that are more stable than their counterparts obtained from other eukaryotes (Hakulinen et al. 2003), making crystallization and other structural studies easier. Our study constitutes the first step to studying its iron binding properties and interactions.

## Materials, methods and results

### Design of the construct

**Fig. 1:**
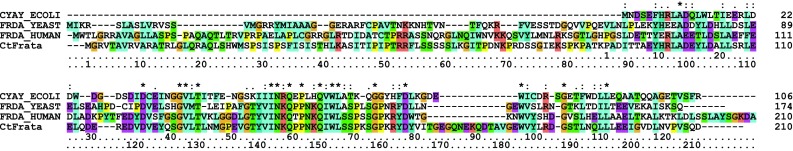
Alignment of the Ct–frataxin sequence with bacterial and eukaryotic frataxins. The alignment was obtained by the ClustalX software. The sequences were retrieved from the pfam database. Stars indicate full conservation, colons strong conservation and commas lower conservation. The two rows below the alignment indicate the numbering of the construct used for this study as also shown in Fig. 2 and the full-length Ct–frataxin numbering

Since Ct–frataxin comes from a eukaryotic organism we had first to predict the domain boundaries to design the construct which would correspond to the C-terminal domain. This was achieved by doing a multiple alignment of prokaryotic and eukaryotic frataxin sequences with the ClustalX software (Fig. 1). The alignment clearly shows a conserved N-terminal region starting around residue A87 which we considered as the start of our construct.

### Protein expression and purification

The recombinant Ct–frataxin C-terminal domain (residues A87-D210) was over-expressed in the *E. coli* host strain BL21 (DE3) using a kanamycin-resistant pet28a LIC vector with a TEV-cleavable His6-GST tag at the N-terminus of the construct. Sole sources of nitrogen and carbon respectively. Isotopically ^15^N- and ^13^C/^15^N-labelled samples were expressed in minimal (M9) medium supplemented with ^15^N-ammonium sulphate and ^13^C-glucose as the sole sources of nitrogen and carbon respectively. Purification was performed using a Ni–NTA agarose column (Qiagen) and the His affinity tag removed by overnight incubation with TEV protease. The protein was further purified by a second Ni-affinity step followed by FPLC size-exclusion chromatography (Superdex 75, GE Healthcare) as previously described (Musco et al. 2000). The concentrations of the Ct–frataxin domain samples were measured by UV absorbance at 280 nm using a calculated extension coefficient of 21,430 M^−1^ cm^−1^ and theoretical molecular weight of 14,130 g mol^−1^ respectively.

### NMR spectroscopy

NMR spectra for resonance assignments were acquired on samples typically containing 1 mM of ^15^N- or ^15^N,^13^C-labelled Ct-frataxin domain. The spectra were recorded at 310.0 K using a Bruker Avance spectrometer operating at 800 MHz ^1^H frequency, equipped with a triple resonance gradient Cold-Probe. All spectra were processed using NMRPipe/NMRDraw software (Delaglio et al. 1995) and analyzed using XEASY (Bartels et al. 1995).

### Resonance assignment and deposition

**Fig. 2:**
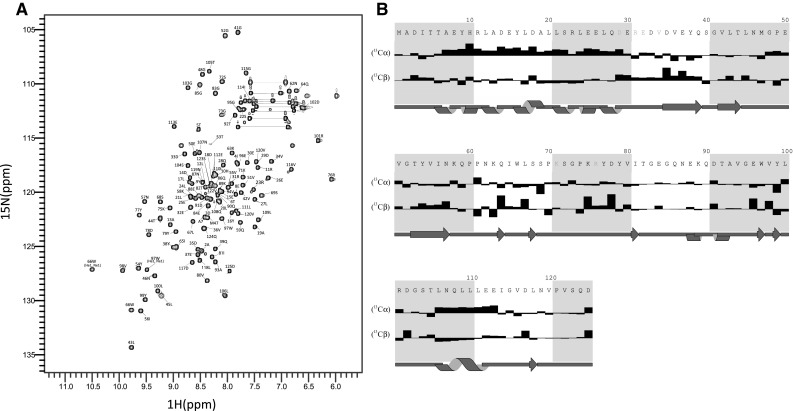
HSQC spectrum and secondary structure derived from NMR assignment. **a** 2D ^1^H, ^15^N-HSQC spectrum of the Ct-frataxin C-terminal domain recorded at 310 K and 800 MHz spectrometer. Side chains of glutamines and asparagines are indicated by a connecting line. **b** Secondary structure as obtained from the chemical shifts. Note that the numbering used refers to the construct and not to the full-length protein

The HSQC of Ct-frataxin is excellent with 125 resonances (Ct–frataxin domain sequence: Ala87–Asp210) clearly defined and resolved (Fig. 2a). Assignment of ^1^H, ^13^C and ^15^N of the Ct–frataxin conserved domain was obtained as described below. Sequence specific assignment of ^1^H, ^15^N, ^13^Cα and ^13^Cβ resonances of the protein were obtained using HNCACB and CBCA(CO)NH experiments (Muhandiram and Kay 1994) in combination with a 2D (^1^H, ^15^N) HSQC experiment. The assignment was carried out using the CCPNMR software (Vranken et al. 2005). Out of the 125 resonances expected in total for the Ct–frataxin domain, we were able to assign all of the HN, ^15^N and ^13^C peaks apart from residue Met1, Ser40 in the 2D HSQC and the Cα resonances of Thr6 and Leu45 (note that this is the construct numbering where the initial Ala2 and the final Asp125 correspond to Ala87 and Asp210 of the gene product). No residues were completely unassigned apart from Pro60 which is connected to Pro61. The secondary structure elements deduced from the assignment are in excellent agreement with what expected from the already known structures (Fig. 2b). The ^1^H, ^13^C and ^15^N chemical shifts of Ct–frataxin have been deposited in the BioMagResBank database and are available under the BMRB Accession Number 27195.
